# Parenteral Nutrition: Amino Acids

**DOI:** 10.3390/nu9030257

**Published:** 2017-03-10

**Authors:** Leonard John Hoffer

**Affiliations:** Faculty of Medicine, McGill University, Montreal, QC H3G 1Y5, Canada; l.hoffer@mcgill.ca; Tel.: +1-514-340-8222

**Keywords:** critical illness, parenteral nutrition, nutritional support, amino acids, protein nutrition

## Abstract

There is growing interest in nutrition therapies that deliver a generous amount of protein, but not a toxic amount of energy, to protein-catabolic critically ill patients. Parenteral amino acids can achieve this goal. This article summarizes the biochemical and nutritional principles that guide parenteral amino acid therapy, explains how parenteral amino acid solutions are formulated, and compares the advantages and disadvantages of different parenteral amino acid products with enterally-delivered whole protein products in the context of protein-catabolic critical illness.

## 1. Nutritional Biochemistry of Amino Acids and Proteins 

### 1.1. Protein and Energy Provided by Amino Acid Mixtures

**Figure 1:**
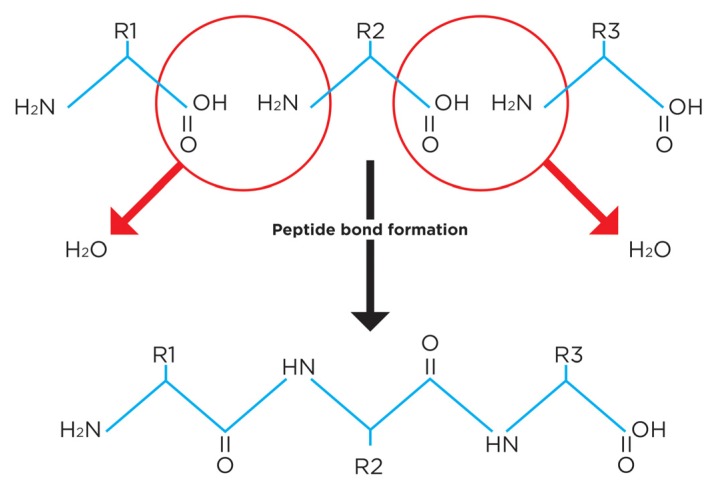
Peptide bond formation is a dehydration reaction. The molecular weight of a free amino acid is greater than its molecular weight in formed protein. Consequently, free amino acids provide less protein substrate and energy than the same weight of formed protein.

Peptide bond formation is a dehydration reaction. For this reason, free amino acids contain less protein substrate, and less energy, than the proteins they create [1]. Figure 1 illustrates why—for the same reason that dextrose provides fewer calories than starch (3.4 versus 4.0 kcal/g)—free amino acids provide less energy and protein substrate than formed protein. For example, 100 g of hydrated mixed amino acids does not provide 400 kcal and 100 g protein, as widely taught, but only 340 kcal and 83 g of protein substrate [1,2].

### 1.2. Essential and Nonessential Amino Acids

Definitions vary, but in general nine of the 21 amino acids required for protein synthesis are termed essential because they cannot be synthesized in the body and hence are required in the diet (histidine, isoleucine, leucine, lysine, methionine, phenylalanine, threonine, tryptophan and valine). Arginine is synthesized, but not always in a sufficient amount; it is considered conditionally essential. Another eight amino acids (alanine, asparagine, aspartate, glutamate, glutamine, glycine, proline—actually an imino acid—and serine) are nonessential because they are readily synthesized from widely available intracellular carbohydrate molecules and amine groups in the large, rapidly interconverting pool of free non-essential free amino acids (NEAAs), predominantly glutamine, glutamate and alanine. The specific contribution of each NEAA to a parenteral amino acid mixture is less important than the total amount of non-essential N supplied. The amino acid mixtures used in parenteral nutrition (PN) compensate for their lack of glutamine (and sometimes glutamate or aspartate) by including sufficiently large amounts of glycine and other NEAAs [2].

Three NEAAs are exceptional: cysteine, selenocysteine and tyrosine. Cysteine is synthesized from methionine, and tyrosine is synthesized from phenylalanine. As long as the diet contains a sufficient amount of methionine or phenylalanine (and the patient is in near-metabolic steady state) cysteine or tyrosine deficiencies should not develop, because these amino acids are on the obligatory catabolic pathways of their corresponding essential amino acid precursors. Metabolic steady state implies that methionine intake equals methionine catabolism, which equals cysteine synthesis. Methionine thus provides both methionine and methionine-derived cysteine. All parenteral amino acid mixtures lack cysteine (it is unstable in solution) although one product includes *N*-acetylcysteine [3]. The activity of a non-rate limiting enzyme of methionine catabolism, cystathionase, is reduced in premature infants. This observation and some indirect clinical evidence, including hypercystathioninemia, suggest that cysteine could become an essential amino acid for premature infants [4]. There is no direct evidence that this temporary, incomplete metabolic blockade is clinically important [2], but some neonatal physicians nevertheless add cysteine hydrochloride to parenteral amino acid mixtures just prior to infusion [5]. The important intracellular reducing agent and reactive metabolic intermediate scavenger, glutathione, is a tripeptide of cysteine, glycine and glutamate. There is conflicting evidence as to whether the provision of cysteine in amounts greater than necessary for protein synthesis beneficially increases erythrocyte glutathione concentrations [3,6]. 

Tyrosine’s concentration in amino acid mixtures is limited by its poor solubility. One product uses the water-soluble tyrosine derivative, *N*-acetylated tyrosine (NAT), instead [3]. This strategy for increasing tyrosine provision comes with a mitigating disadvantage, namely, that humans de-acetylate NAT slowly and their renal tubules reabsorb it inefficiently [7]. One may speculate that loss of NAT in the urine could become a problem in diseases associated with aminoaciduria, such as critical illness, in which a 5-fold increase in urinary free tyrosine excretion has been reported [8]. Tyrosine deficiency seems unlikely, however, because as long as phenylalanine provision is sufficient, the patient is in neutral or negative N balance and phenylalanine conversion to tyrosine unimpeded, the tyrosine requirement will be met by phenylalanine alone.

## 2. Standard and Specialized Amino Acid Mixtures

Standard amino acid solutions contain all nine essential amino acids in patterns and amounts designed to match or exceed their recommended dietary allowances, sufficient amine N from some NEAAs to support the synthesis of all them, and a generous amount of arginine [3,9,10,11]. The recommended normal adult protein requirement is 0.8 g/kg per day. To meet this requirement a parenteral amino acid solution must be infused at the rate of ~1.0 g/kg per day. 

Specialized amino acid solutions are available for patients with hepatic insufficiency (increased amounts of branched-chain amino acids and less methionine, phenylalanine and tryptophan), renal insufficiency (essential amino acids largely or only), and protein-catabolic critical illness (increased branched-chain amino acids). In some countries a commercial solution of 20 g/100 mL alanyl-glutamine is available to treat presumed glutamine deficiency states [3,9,10]. None of these products has clearly been shown to be superior to standard parenteral amino acid therapy [12,13]. They are not further considered in this review, except to suggest that the claim that glutamine is a conditionally essential amino acid is questionable, given the lack of evidence that glutamine synthesis is impaired when its substrate (all other amino acids) is provided in a sufficient amount. What has been termed nutritional glutamine deficiency may, at least in part, represent inadequate total protein provision to protein-catabolic patients [2]. 

## 3. Premixed versus Individually Compounded Amino Acid Mixtures 

Many hospital pharmacies purchase premixed ready-to-use PN products that are either dual-chamber (amino acids and dextrose, with or without electrolytes) or triple-chamber (amino acids, dextrose and lipid). Shortly prior to infusion, the internal membranes separating the chambers are broken, their contents intermixed and vitamins, trace minerals and additional electrolytes added [14,15]. These products are convenient and potentially cost-effective, but their fixed nutrient composition is a drawback. They are commonly provided in volumes calculated to match the patient’s calorie requirement, with resulting under-provision of amino acids [14]. A variety of dual-chamber amino acid/dextrose products are available, so—at least when the protein requirement does not exceed ~1.5 g/kg per day—it is possible to meet a patient’s protein requirement and avoid calorie overfeeding by selecting an appropriate fixed-composition product. The downside of this approach is the inconvenience of stocking the pharmacy with many different premixed products. In some situations—especially critical illness, in which 2.5 g/kg protein substrate per day may be required—a computer-controlled sterile compounder is necessary. This instrument combines stock amino acid solutions (15 to 20 g/100 mL) and dextrose (70 g/100 mL) to create mixtures of amino acids and dextrose that precisely meet both the protein and calorie requirements of individual patients, and in a much smaller volume. A later section of this article illustrates the advantages of this approach. 

## 4. Effects of Starvation and Disease on Protein Metabolism and Requirements 

Dietary protein and energy deficiency are common in acutely hospitalized patients, and universal in critical illness. Inattention to these nutritional deficiencies leads to the disease called protein-energy malnutrition, the cardinal features of which are generalized muscle atrophy and fat loss. Muscle atrophy is much more dangerous than fat loss, since it is life-threatening, debilitating and difficult to reverse, whereas the great majority of modern hospitalized patients have adequate (or more than adequate) fat reserves to draw upon during temporary periods of hypocaloric nutrition. 

In addition to the muscle atrophy caused by deficient dietary protein and energy provision, many diseases or their treatments increase dietary protein requirements well above normal by increasing body amino acid or protein loss (in wound exudates or fistulas, inflammatory diarrhea, and renal replacement therapy) or by pathologically increasing muscle protein catabolism, as occurs with high-dose glucocorticoid therapy and as part of the systemic inflammatory response to sepsis and major trauma.

### 4.1. Protein and Energy Requirements in Critical Illness

For more than four decades there has been a consensus—based on consistent, convincing animal and human metabolic data, N balance studies and clinical observational data—that critical illness increases human protein requirements [16,17,18,19,20,21,22,23,24]. Regrettably, suitably-powered hard clinical outcome trials to confirm or refute this physiologically plausible conclusion have not yet been carried out [2]. Instead, until recently, large clinical nutrition trials in critical illness focused on the benefits of calorie supplementation, while largely ignoring or dismissing the importance of protein or amino acids. The results have been very disappointing. The flawed logic of this calorie-focused approach has been analyzed elsewhere [22,25,26]. The hypothesis that continues to await rigorous testing is that prompt, high-protein (2–2.5 g/kg per day) hypocaloric nutrition may improve clinical outcomes in catabolic critical illness.

### 4.2. Appropriate Dose of Energy and Protein in Critical Illness

Pending the results of future clinical trials, I suggest that energy provision be restricted to ~2/3 of a non-fat-depleted patient’s rate of energy expenditure [27]. This recommendation is based on three arguments:

(1) The protein-wasting effect of hypocaloric (low carbohydrate) nutrition is minor to minimal at energy provision rates greater than ~50% of energy expenditure, and can be mitigated by increasing protein provision [27].
(2) The enteral regimens currently used in critical care are hypocaloric and protein-deficient. Thanks to many recent clinical trials, we may now conclude that short-term hypocaloric nutrition does not worsen clinical outcomes (and could even be beneficial) for patients with a body mass index >17 kg/m^2^ [22,27,28].
(3) Energy expenditure estimation is so inexact, calorie overfeeding so toxic, and short-term calorie underfeeding so benign, that prudence dictates erring on the side of hypocaloric nutrition for the great majority of critically ill patients.

Existing physiological, observational, and biomarker evidence suggests that early protein provision should be increased to somewhere between 1.5 and 2.5 g protein/kg normalized dry body weight per day [16,17,18]. Physiological reasoning predicts that patients experiencing more intense protein catabolism, as indicated by a high rate of urinary N excretion in relation to their existing muscle mass, are most likely to benefit from suitably generous protein provision [29].

### 4.3. Promptness of Amino Acid Provision

The clinical outcome of septic shock appears to be greatly improved by very prompt, appropriate antimicrobial and prophylactic heparin therapy [30,31]. The same general logic applies to nutrition therapy. Prompt generous protein provision could improve clinical outcomes in the early, potentially most modifiable phase of critical illness by mitigating the rapid muscle atrophy that accompanies it and supplying the tissues with the amino acids required for optimum wound healing and host defense. 

Against this view, two articles have suggested that early protein provision is dangerous in critical illness. One of them, citing a post-hoc analysis of a clinical trial, concluded that patients receiving less protein had better clinical outcomes [32]. Post-hoc analysis of this kind is notoriously untrustworthy, and it is especially suspect here because all the protein intakes in the clinical trial were well below a physiologically plausible threshold either for benefit or harm. Moreover, as DK Heyland has pointed out, the divergence in primary clinical outcome rates attributed to the amino acids in the parenteral amino acid-supplemented arm of this trial occurred before parenteral amino acid provision commenced (DK Heyland, personal communication). Another, small observational trial similarly led its authors to conclude that a higher level of protein provision may worsen outcomes in critical illness [33,34]. An exchange of correspondence subsequently revealed that even the highest level of protein provision in that study fell well below a plausible threshold for benefit or harm [35]. 

As with protein provision in general, there is no pertinent clinical trial evidence available either to support or refute the hypothesis that prompt generous protein provision is beneficial in critical illness. It is important to test this hypothesis, because critically ill patients currently receive less than half the currently recommended amount of protein—less even than a healthy person’s requirement—for at least the first two weeks of treatment in modern intensive care units (ICUs) [22].

## 5. Enteral Protein versus Parenteral Amino Acids 

EN is preferred to PN for invasive nutrition support because of its simplicity, trophic effects on the gastrointestinal tract and a possibly reduced risk of infectious complications. PN is more resource-intensive, potentially riskier, and requires somewhat more expertise than EN. It is indicated when invasive nutrition support is required and EN is refused, inappropriate, or demonstrated (or strongly predicted) to be incapable of meeting the patient’s nutritional needs. The major disadvantage of EN in critical illness is its slowness. EN commonly fails to achieve the patient’s nutritional goal—especially for protein—within the first 7–14 days of therapy [22]. PN’s chief advantage is its ability to deliver a substantial dose of amino acids very promptly [36]. PN’s safety concerns are inadvertent calorie overfeeding, potential amino acid over-provision, and a possibly greater risk of infectious complications. However, the differences reported in complication rates between EN and PN reflect observations from older studies when calorie overfeeding was routine [21,37].

### Maximum Dose of Parenteral Amino Acids

The key advantage of parenteral amino acid solutions—almost immediate infusion of a substantial amount of protein substrate without other calorie sources—is mitigated by the potential for amino acid over-provision. The most obvious way protein can be toxic is by generating ammonia in excess of the urea cycle’s capacity. Hyperammonemia and encephalopathy are complications of protein or amino acid provision in severe liver dysfunction, heterozygous ornithine transcarbamoylase deficiency or other urea cycle disorders. Patients with overwhelming critical illness and hepatic hypoperfusion are at risk of hyperaminoacidemia even in the absence of amino acid infusions [16].

Some authors regard azotemia (an increased plasma urea concentration) as evidence of amino acid toxicity, but do not explain why they so regard it in patients not requiring renal replacement therapy. Urea synthesis is increased in critical illness and further increased in proportion to the rate of protein provision, but plasma urea concentrations are far more greatly influenced by renal blood flow and renal functional mass than the rate of protein provision. The available evidence suggests that protein provision in doses between 2.5 and 3.0 g/kg normal weight day are safe for use in clinical trials and prudent clinical practice except in patients with refractory hypotension, overwhelming sepsis or serious liver disease [16].

## 6. Selection and Dosage of Enteral and Parenteral Products 

We have argued elsewhere that investigation of the optimum protein requirement in critical illness has lapsed for decades because clinical investigators ignored the principles of physiological nutrition [20,38]. A cognate reason is inattention and, presumably, the lack of a commercial market for high-protein, energy-restricted enteral products by the nutrition industry. Until very recently, there was a lack of easy-to-use EN products capable of delivering generous amounts of protein or amino acids without energy overfeeding. 

Fortunately, several high-protein, energy-restricted EN products are now on the market. Let’s compare their performance with high amino-acid, low-dextrose PN. Three issues should be considered when selecting a route of nutrient administration (EN versus PN) and product: (1) the maximum dose of protein it can provide without calorie overfeeding; (2) the number of days required to reach the protein goal; and (3) the volume of product required to deliver it, in view of the important problem of fluid volume overload in the ICU [39]. 

Table 1 lists several available EN and PN products and indicates the amounts of protein (or protein substrate in the case of amino acids) and energy contained in one liter of the product. Table 2, which follows Table 1 and was created from the data in it, indicates the calorie dose and volume required to provide a hypothetical protein-catabolic critically ill patient whose dry body weight is 70 kg with either of two biologically plausible protein doses: 1.5 g/kg (105 g) or 2.0 g/kg (140 g). (It should be noted that the addition of electrolytes, vitamins and trace elements to the PN admixture increases the total volume by ~200 mL).

The data in Table 2 support three conclusions.

(1) Most standard premixed PN products deliver too much energy, volume, or both to be safe or practical in critical illness.
(2) High-protein energy-restricted EN products are capable of providing target protein doses, although in some instances require a large fluid volume. The number of days required to reach these targets remains an open question.
(3) Compounded PN solutions easily and almost immediately provide target amounts of protein substrate and very few calories. In the most striking example, sole use of the product with the maximum amino acid concentration (20 g/100 mL) can provide 140 g protein substrate and only 560 kcal in ~900 mL.

## 7. A Practical Clinical Scenario

Current standard ICU nutrition—known as “permissive underfeeding” (of protein, calories and micronutrients) for the first week or two of an ICU stay—is not based on evidence of its merit. It is, rather, the unintended consequence of recommendations to avoid PN and rely solely on low-protein EN that progresses very slowly toward the target calorie dose. We now know that short-term hypocaloric nutrition is safe; the “hypocaloric” part of permissive underfeeding is therefore not a problem. We are increasingly confident that high-amino acid, hypocaloric PN is safer than traditional high-calorie PN. EN products are now available with a protein-to-calorie ratio that’s suitable for critically ill patients. It remains to be demonstrated that these EN products can deliver target protein doses promptly enough. Prompt high-protein, hypocaloric nutrition support, while physiologically plausible, remains untested in suitable clinical trials. 

Pending the outcome of such trials, clinicians may choose prompt high-protein hypocaloric nutrition, especially for patients whose rate of urinary N excretion indicates a high rate of muscle protein catabolism. Premixed PN solutions are not appropriate for this purpose, for they deliver too much dextrose or too much volume. Modern, high-protein EN products may prove to be satisfactory. Compounded PN is able to provide very large doses of amino acids without calorie overfeeding or volume overload. An obvious clinical scenario is to simultaneously commence high-protein EN and full-dose parenteral amino acids (without dextrose), then progressively reduce the parenteral amino acid dose as protein delivery by EN gradually increases to reach the target. 

The disadvantage of simultaneous EN and high amino-acid low-energy PN is that it’s more complicated, and complexity can bring complications. Whether or not the disadvantages of this regimen outweigh its advantages can only be found out by designing and carrying out physiologically literate clinical trials. For now, it’s a plausible strategy for clinical trial design, and safe and plausible enough for empirical clinical care by knowledgeable clinicians.

## Figures and Tables

**Table 1:** Protein and energy content of some high-protein energy-restricted parenteral and enteral nutrition products.

Manufacturer	Product	Route	Protein Substrate (g/L)	Dextrose/CH_2_O ^1^ (g/L)	Lipid (g/L)	Energy (kcal/L)	Comment
B Braun	Nutriflex	Parenteral	70 g amino acids	240 (dextrose)	0	1054	Dual-chamber
	7%/24% ^2^		(58 g protein)				
Baxter	Clinimix	Parenteral	50 g amino acids	50 (dextrose)	0	340	Dual-chamber
	5%/5%		(42 g protein)				
Fresenius Kabi	Aminomix	Parenteral	50 g amino acids	120 (dextrose)	0	578	Dual-chamber
	5%/12%		(42 g protein)				
Pfizer	Aminosyn II	Parenteral	42.5 g amino acids	100 (dextrose)	0	483.	Dual-chamber
	4.25%/10%		(36 g protein)				
Pfizer	Aminosyn II	Parenteral	35 g amino acids	50 (dextrose)	0	289	Dual-chamber
	3.5%/5%		(29 g protein)				
Various manufacturers	Compounded	Parenteral	120 g amino acids	140 (dextrose)	0	884	From 15% amino acids and 70% dextrose
	12%/14% ^3^		(100 g protein)				
Baxter	Prosol 20%	Parenteral	200 g amino acids	0	0	664	
			(166 g protein)				
Abbott	Vital High Protein	Enteral	87.5 g protein	112 (CH_2_O)	23	1000	
Fresenius Kabi	Fresubin Intensiv	Enteral	100 g protein	129 (CH_2_O)	32	1220	
Nestle	Peptamen Intense High Protein	Enteral	93.2 g protein	78 (CH_2_O)	38	1000	

^1^ CH_2_O, carbohydrate; ^2^ Amino acids/dextrose (g/100 mL); ^3^ Illustrative example.

**Table 2:** Energy and Volume Delivered to Provide Protein or Protein Substrate to a 70 kg Patient.

Manufacturer	Product	Route	105 g Protein or Protein Substrate		140 Protein or Protein Substrate	
			Energy (kcal)	Volume (mL)	Energy (kcal)	Volume (mL)
B Braun	Nutriflex	Parenteral	1908	1810	2544	2414
	7%/24% ^1^					
Baxter	Clinimix	Parenteral	850	2500	1133	3333
	5%/5%					
Fresenius Kabi	Aminomix	Parenteral	1445	2500	1927	3333
	5%/12%					
Pfizer	Aminosyn II	Parenteral	1409	2917	1878	3889
	4.25%/10%					
Pfizer	Aminosyn II	Parenteral	1046	3621	1395	4828
	3.5%/5%					
Various manufacturers	Compounded	Parenteral	928	1050	1238	1400
	12%/14% ^2^					
Baxter	Prosol 20%	Parenteral	420	633	560	843
Abbott	Vital High Protein	Enteral	1200	1200	1600	1600
Fresenius Kabi	Fresubin Intensiv	Enteral	1281	1050	1708	1400
Nestle	Peptamen Intense High Protein	Enteral	1127	1127	1517	1517

^1^ Amino acids/dextrose (g/100 mL); ^2^ Illustrative example.
